# P-1964. Pre-exposure Prophylactic Treatment with Ensitrelvir Inhibited SARS-CoV-2 Infection in Hamster Aerosol Transmission Model

**DOI:** 10.1093/ofid/ofae631.2123

**Published:** 2025-01-29

**Authors:** Masaaki Nakashima, Haruaki Nobori, Takayuki Kuroda, Alice Shimba, Satoshi Miyagawa, Akane Hayashi, Kazumi Matsumoto, Kaoru Baba, Keita Fukao

**Affiliations:** Shionogi&Co., Ltd., Toyonaka, Osaka, Japan; SHIONOGI & CO., LTD., Toyonaka-shi, Osaka, Japan; Shionogi&Co., Ltd., Toyonaka, Osaka, Japan; Shionogi&Co., Ltd., Toyonaka, Osaka, Japan; Shionogi&Co., Ltd., Toyonaka, Osaka, Japan; Shionogi TechnoAdvance Research, Co., Ltd., Toyonaka, Osaka, Japan; Shionogi TechnoAdvance Research, Co., Ltd., Toyonaka, Osaka, Japan; Shionogi TechnoAdvance Research, Co., Ltd., Toyonaka, Osaka, Japan; Shionogi&Co., Ltd., Toyonaka, Osaka, Japan

## Abstract

**Background:**

Although aerosol virus transmission is the major transmission route for SARS-CoV-2 and there remains a need to control SARS-CoV-2 spread with antivirals, prophylactic effect of pre-exposure antiviral treatments to prevent SARS-CoV-2 aerosol transmission remains to be clarified. In a previous report, we established the SARS-CoV-2 aerosol transmission hamster model (ESWI 2023, Nakashima et al.) (Figure 1). Here, we administered ensitrelvir to naive hamsters (contact) prior to exposure of virus aerosol shed by virus-infected hamsters (index) and evaluated the efficacy of the antivirals on SARS-CoV-2 aerosol infection.

**Figure 1:**
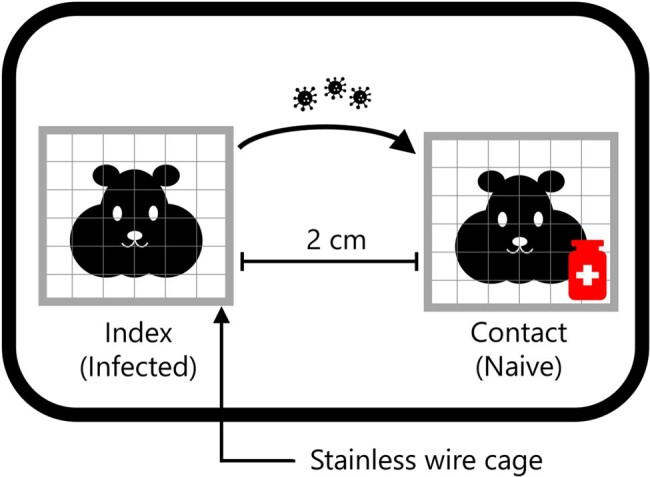
Experimental aerosol SARS-CoV-2 transmission hamster model

**Methods:**

Six syrian hamsters were intranasally inoculated with SARS-CoV-2 delta strain (index). Six naïve syrian hamsters (contact) were administered subcutaneously ensitrelvir 250 or 750 mg/kg twelve hours before the cohousing based on ensitrelvir exposure in human. Index and contact hamsters were cohoused in separated two stainless wire cages to prevent direct viral transmission, and in same container allowing aerosol transmission of SARS-CoV-2 for twelve hours on one day post infection. To evaluate viral titers in lung and nasal lavage fluid (NALF), both samples were collected from index hamsters two days post infection and from contact hamsters four days after cohousing, respectively. To evaluate pathogenesis, body weight changes were monitored until ten days post infection and lung weights were measured ten days post infection.

**Figure 2:**
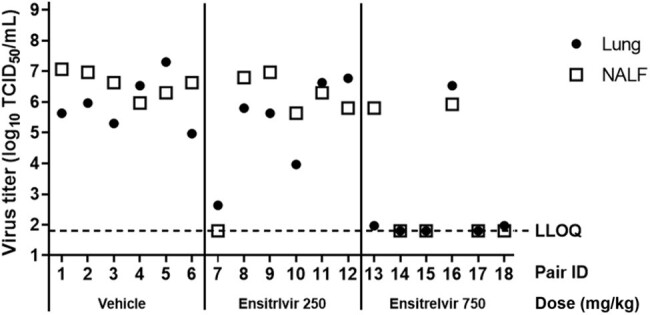
The effect of prophylactic treatment with ensitrelvir on the viral titers in lungs and NALF in contact hamsters TCID50: median tissue culture infectious dose, NALF: nasal lavage fluid, LLOQ: Lower limit of quantification

**Results:**

Virus was detected in the lung and NALF of all contact hamsters administered with vehicle, demonstrating efficient aerosol transmission. Pre-exposure prophylactic treatment of contact hamsters with ensitrelvir 750 mg/kg reduced the aerosol transmission (Figure 2). Furthermore, pre-exposure treatment of 750 mg/kg ensitrelvir surpassed body weight loss and lung weight increase of aerosol infected hamsters compared to vehicle-treated hamsters.

**Conclusion:**

Pre-exposure treatment of hamsters with ensitrelvir prevented hamsters from aerosol virus infection and suppressed body and lung weight changes in hamster SARS-CoV-2 aerosol transmission model. This approach may help protect at-risk individuals who live or work with SARS-CoV-2-infected patients.

**Disclosures:**

Masaaki Nakashima, PhD, Shionogi&Co., Ltd.: Employee|Shionogi&Co., Ltd.: Stocks/Bonds (Private Company) Haruaki Nobori, PhD, Shionogi & Co., Ltd.: Employee|Shionogi & Co., Ltd.: Stocks/Bonds (Private Company) Takayuki Kuroda, n/a, Shionogi & Co., Ltd.: Employee|Shionogi & Co., Ltd.: Stocks/Bonds (Private Company) Alice Shimba, n/a, Shionogi & Co., Ltd.: Employee|Shionogi & Co., Ltd.: Stocks/Bonds (Private Company) Satoshi Miyagawa, n/a, Shionogi & Co., Ltd.: Employee|Shionogi & Co., Ltd.: Stocks/Bonds (Private Company) Akane Hayashi, n/a, Shionogi & Co., Ltd.: Stocks/Bonds (Private Company)|Shionogi TechnoAdvance Research: Employee Kazumi Matsumoto, n/a, Shionogi TechnoAdvance Research: Employee Kaoru Baba, n/a, Shionogi TechnoAdvance Research: Employee Keita Fukao, MD, SHIONOGI and CO., LTD.: Stocks/Bonds (Private Company)

